# Efficacy of an automated technology at detecting early postpartum estrus events: Can we detect resumption of cyclicity?

**DOI:** 10.3168/jdsc.2023-0463

**Published:** 2023-11-17

**Authors:** S. Borchardt, T.A. Burnett, W. Heuwieser, J.L. Plenio, R.S. Conceição, R.L.A. Cerri, A.M.L. Madureira

**Affiliations:** 1 Clinic of Animal Reproduction, Freie Universität Berlin, 14163, Berlin, Germany; 2 University of Guelph, Ridgetown Campus, Ridgetown, ON, Canada N0P 2C0; 3 Institute for Veterinary Epidemiology and Biostatistics, Freie Universität Berlin, 14163, Berlin, Germany; 4 Faculty of Land and Food Systems, University of British Columbia, Canada V6T 1Z4

## Abstract

The objective of this observational study was to evaluate the efficacy of a neck-mounted automated activity monitor (AAM) at detecting early postpartum resumed ovarian cyclicity. A total of 192 lactating cows (primiparous = 73 and multiparous = 119) were enrolled in this study. Cows were continuously monitored by a neck-mounted AAM early postpartum (7 to 30 d in milk; DIM). Calving was classified as assisted (forced extraction of a calf) or unassisted (normal calving). Retained fetal membrane, metritis, hyperketonemia, clinical mastitis, and milk production were recorded. Cows were classified as healthy (i.e., no disease events) or sick (i.e., any disease event). Estrus events were alerted by the AAM using a proprietary algorithm set by the AAM company. Blood samples, from the coccygeal vein, were collected at 15, 18, 21, 24, 28, and 30 DIM for progesterone (P4) analysis. Resumption of cyclicity was considered when P4 concentration was ≥1 ng/mL on any collection day. Cows were considered anovular when P4 concentration was <1 ng/mL on all collection days. Cows were classified as true positive: P4 ≥ 1 ng/mL and at least one estrus alert; false positive: P4 < 1 ng/mL and at least one estrus alert; true negative: P4 < 1 ng/mL and no estrus alerts; and false negative: P4 ≥ 1 ng/mL and no estrus alerts. Statistical analyses were performed by frequency distribution and mixed effects logistic regression procedures on SAS (SAS Institute Inc.). The specificity, sensitivity, accuracy, and positive predictive value of the sensor to detect cows that had resumed cyclicity were 84.0%, 34.1%, 52.1%, and 79.2%, respectively. Out of the 192 cows, 35.9% (69/192) were anovulatory and 37.5% (72/192) had no estrus events between 7 to 30 DIM. Healthy cows were more likely to resume cyclicity in early lactation compared with cows that were sick (78.3 ± 1.9 vs. 32.8 ± 3.1%, respectively) independent of parity. In conclusion, the sensor had a high specificity for detecting anovular cows, but it had lower sensitivity, and thus was not effective at detecting cyclic cows, perhaps due to silent ovulation early postpartum.

## Graphical Abstract

**Figure:**
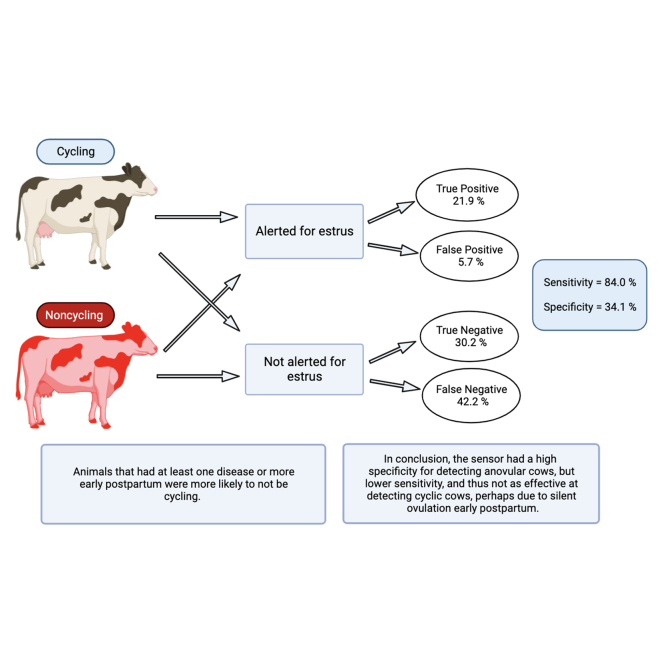

Resumption of regular cyclicity after calving is a crucial aspect of reproductive health in dairy cattle. It directly influences reproductive performance and calving intervals, affecting the overall profitability of dairy operations (Walsh et al., 2007; Galvão et al., 2013). Assessment of anovulation before the end of the voluntary waiting period (**VWP**) is labor intensive, requiring multiple examinations either by analyzing circulating progesterone (**P4**) concentrations or by visualization of a corpus luteum using transrectal ultrasound (Lucy, 2019). Therefore, it has not been adopted on commercial dairy farms. In recent years, advancements in technology have led to the development of automated activity monitoring (**AAM**) systems, providing valuable insights into the reproductive status of dairy cows. By enabling early detection of estrus, monitoring cow health, and providing data-driven decision-making, AAM contribute to improve conception rates, reduce calving intervals, and optimize herd productivity.

Recently, an approach that consists of identify a subgroup of cows that need a specific reproductive management strategy to achieve better reproductive performance has been described and named targeted reproductive management (Giordano et al., 2022). As technology continues to advance, incorporating traditionally unused AAM data (e.g., occurrence of estrus within the VWP) into targeted reproductive management may contribute to increased sustainability and profitability of dairy farms (Rial et al., 2022; Gonzalez et al., 2023).

Numerous studies have been conducted to evaluate the efficiency and reliability of AAM to identify animals in estrus. The percentage of animals accurately detected using AAM varies between 71% and 90% both in confinement and pasture-based systems (Aungier et al., 2015; Dolecheck et al., 2015). While several AAM systems have been validated to identify estrus alerts and ovulation in lactating dairy cows, none of these studies used cows before the VWP. The detection of the first ovulation might be advantageous, but more difficult for the sensor as the first ovulation after parturition is often described as a silent ovulation without obvious estrus behavior (Crowe et al., 2014; Borchardt et al., 2021).

Therefore, the objective of this observational study was to evaluate the efficacy of a neck-mounted AAM system to identify ovulation in lactating dairy cows from 7 to 30 DIM. Our hypotheses were that an AAM system can be used to detect ovulatory status within the VWP and therefore can be used to facilitate targeted reproductive management.

This observational study was conducted on one commercial farm in Slovakia, from June 2022 to August 2022. The local Institutional Animal Care Committee, following the requirements of the practices outlined in the *Guide for the Care and Use of Agricultural Animals in Research and Teaching* (FASS, 2020), approved all experimental procedures. Lactating cows were housed in a naturally ventilated freestall barn and were fed twice daily with a TMR that was formulated to meet or exceed the requirements of a lactating Holstein cow (NRC, 2001). Water and TMR were available ad libitum.

Cows were screened for health disorders based on visual observations performed by a trained researcher. Calving assistance during parturition was carried out by farm personnel based on the discretion of farm management. Assisted calving was defined as forced extraction of a calf via manual pulling or use of calf chains from one or more farm personnel. Metritis (**METR**), retained fetal membrane (**RFM**), hyperketonemia (**KET**), left displaced abomasum (**LDA**), and clinical mastitis (**MAST**) until 30 DIM were recorded.

Vaginal discharge (**VD**) was evaluated for each cow at 3, 5, 8 and 13 d postpartum, using a Metricheck device (Simcrotech) that was inserted into the vaginal canal up to the cervix. The appearance and smell of the VD was evaluated using the following scale adapted from Williams et al. (2005): 1 = no discharge or clear mucus; 2 = cloudy mucus with <50% purulent; 3 = purulent >50%; and 4 = putrid (red or brown color, watery, foul smelling). Cows were classified as having METR if they had at least one VD score of 4. Cows with RFM after 24 h after calving were diagnosed as having an RFM. Hyperketonemia was assessed at 3, 8, and 13 d postpartum. Blood ketone concentration was evaluated using a cow-side electronic handheld device (TaiDoc, Pharmadoc; validated by Bach et al., 2016). Hyperketonemia was defined as whole blood BHB concentrations ≥1.2 mmol/L at one or more of the 3 postpartum blood samples (McArt et al., 2013). Cows were considered to suffer from LDA when percussion of the left flank resulted in tympanic resonance auscultated with a stethoscope. Clinical mastitis was characterized by an altered milk secretion, which includes the appearance of clots, flakes, discoloration, or abnormal consistency, as well as apparent indicators of inflammation in an affected mammary gland, such as redness, swelling, pain, or heat. The health of the udder was examined by farm workers twice daily during routine milking times. All treatments for METR, RFM, KET, LDA, and MAST were based on farm management and made by the on-site herd veterinarian using standardized protocols.

Twenty-one days before their first calving, all cows were equipped with a neck-mounted AAM system (Smarttag Neck, Nedap Livestock Management) validated by Roelofs et al. (2017). When the activity exceeds a threshold for multiple consecutive periods, a cow is considered in estrus and an AAM alert (i.e., attention) is generated. Data for each estrus early postpartum (7 to 30 DIM) were recorded.

Blood samples were collected from the coccygeal vein on d 15, 18, 21, 24, 28, and 30 postpartum for the analysis of P4. Blood was extracted into a commercial blood collection tube with EDTA (8 mL, Vacuette, Greiner Bio-One GmbH), and centrifuged after collection at 2,700 × *g* for 15 min at approximately 20°C. Plasma was stored at −18°C until shipment to a commercial laboratory (Segalab, accreditation number L0295). Plasma P4 concentrations were determined, using an enzyme-labeled chemiluminescent competitive immunoassay (Immulite Progesterone Enzym, Siemens Healthcare). Intra- and interassay coefficients of variation for 20 assays for repeated samples averaged 7.35 ± 2.44% and 8.08 ± 2.42%, respectively. The lower limit of detection was 0.2 ng/mL.

Sample size (n = 175) was determined following the approach proposed by Hajian-Tilaki (2014) using a minimum sensitivity of 0.8 and a minimum specificity of 0.7, and 95% confidence and 80% power. Before all analyses, continuous dependent variables were checked for normality using the UNIVARIATE procedure and probability distribution plots. Means, standard deviations, distributions, and normality tests were obtained using the UNIVARIATE procedure of SAS University Edition (SAS Institute Inc., Cary, NC). Parity was classified into primiparous (first lactation) and multiparous (≥second lactation). Resumption of cyclicity was considered when P4 concentration was ≥1 ng/mL at any collection day. Cows were considered anovular when P4 concentration was <1 ng/mL on all collection days. Progesterone concentrations were used as a gold standard to calculate the test characteristics of the AAM system. Cows were classified as true positive: P4 ≥1 ng/mL and at least one estrus alert; false positive: P4 <1 ng/mL and at least one estrus alert; true negative: P4 <1 ng/mL and no estrus alerts; and false negative: P4 ≥1 ng/mL and no estrus alert. Equations used to determine the precision (i.e., sensitivity, specificity, accuracy, positive predictive value, and negative predictive value) of the AAM to detect cyclic cows are shown in Table 1. Cows were classified according to the incidence of disease early lactation; cows with no episodes of diseases were classified as healthy and cows that had one or more diseases during the period of the data collection were classified as having disease (one or more diseases during the period of data collection).

**Table 1:** Equations used to determine the test characteristics of an automated activity monitor to identify cows that resumed ovarian cyclicity in early lactation (7 to 30 DIM) based on plasma progesterone concentration

Test characteristic	Equation^1^
Sensitivity	TP/(TP + FN) × 100
Specificity	TN/(TN + FP) × 100
Accuracy	[(TP + TN)/(TP + TN + FP + FN) × 100]
Positive predictive value	TP/(TP+FP) × 100
Negative predictive value	TN/(TN+FN) × 100

1 True positive (TP) = progesterone ≥1 ng/mL and at least one estrus alert. True negative (TN) = progesterone <1 ng/mL and no estrus alerts. False positive (FP) = progesterone <1 ng/mL and at least one estrus alert. False negative (FN) = progesterone ≥1 ng/mL and no estrus alerts.

Frequency distribution of disease, false negatives, false positives, true negatives, and true positives were summarized and tested using chi-square on PROC FREQ procedure from SAS. The resumption of cyclicity was used as a binomial dependent variable and tested for the effects of lactation (primiparous and multiparous), calving ease, RFM, KET, METR, LDA, and MAST, using mixed effects logistic regression using the GLIMMIX procedure by specifying the distribution as binary and cow as the experimental unit. Marginal means were obtained from the mixed logistic regression model. All explanatory variables were checked for collinearity, where categorical variables were assessed using contingency tables; no multicollinearity of explanatory variables were found.

For all models, only variables with a *P*-value <0.15 were kept in final models, using manual backward elimination. Differences between groups with *P* ≤ 0.05 were considered significant and those between 0.05 > *P* ≤ 0.10 were designated as a tendency, when using Tukey's adjustments for multiple comparisons. Interactions were tested between all variables selected in the final model and kept if *P* < 0.05.

A total of 192 lactating dairy cows (primiparous = 73 and multiparous = 119) were enrolled in the study. Mean (± SD) lactation was 2.2 ± 1.3 and cows were producing 40.5 ± 12.0 kg/d between 7 to 30 DIM.

The total numbers of cows demonstrating false negative, false positive, true negative, and true positive alerts were 81, 11, 58, and 42, respectively, and these did not differ between primiparous and multiparous cows (false negative, *P* = 0.95; false positive, *P* = 0.90; true negative, *P* = 0.52; and true positive, *P* = 0.48). The test characteristics of the AAM are shown in Table 2. No differences were detected in test characteristics of the AAM between multiparous and primiparous cows. Overall, the specificity and sensitivity of the sensor to detect cyclic cows early postpartum were 84.0% and 34.1%, respectively (Table 2). Out of the 192 cows, 35.9% (69/192) were anovular based on P4 (<1 ng/mL); however, 62.5% (120/192) of cows did not have any alerts detected by the AAM for estrus event between 7 to 30 DIM.

**Table 2:** Test characteristics of an automated activity monitor [% (95% CI)] to identify cows that resumed ovarian cyclicity in early lactation (7 to 30 DIM) based on plasma progesterone concentration (n = 192) according to parity (multiparous [n = 119] and primiparous [n = 73])

Test characteristic^1^	Overall	Multiparous^2^	Primiparous^3^
Sensitivity	34.1	35.9	31.1
	(25.8–43.2)	(25.3–47.5)	(18.2–46.6)
Specificity	84.0	82.9	85.7
	(73.3–91.8)	(67.9–92.8)	(67.3–95.9)
Accuracy	52.1	52.1	52.0
	(44.8–59.3)	(42.7–61.3)	(40.0–63.9)
Positive predictive value	79.2	80.0	77.8
	(67.8–87.4)	(65.7–89.3)	(56.1–90.5)
Negative predictive value	41.7	40.5	43.6
	(37.8–45.7)	(35.4–45.8)	(36.7–49.8)

1 Sensitivity = TP/(TP + FN) × 100. Specificity = TN/(TN + FP) × 100. Accuracy = [(TP + TN)/(TP + TN + FP + FN) × 100]. Positive predictive value = TP/(TP+FP) × 100. Negative predictive value = TN/(TN+FN) × 100. TP = true positive; TN = true negative; FP = false positive; FN = false negative.

2 Multiparous: ≥third lactations.

3 Primiparous: first lactation.

The incidence of metritis was 10.9% (21/192); primiparous cows had a greater incidence of metritis compared with multiparous (17.8% [13/73] vs. 6.7% [8/119]; *P* = 0.01, respectively). Multiparous cows had more assisted calvings than primiparous (35.3% [42/119] vs. 17.8% [13/73]; *P* < 0.01, respectively). The incidence of hyperketonemia was 22.4% (43/192) where multiparous cows had a greater percentage of hyperketonemia compared with primiparous cows (34.5% [41/119] vs. 2.7% [2/73], *P* < 0.01, respectively). No differences were found between multiparous and primiparous cows regarding RFM (*P* = 0.22) and MAST by 30 DIM (*P* = 0.62). Healthy cows were more likely to resume cyclicity in early lactation compared with cows that had diseases (78.3 ± 1.9% vs. 32.8 ± 3.1% *P* = 0.03, respectively), independent of parity.

The objective of this observational study was to determine the test characteristics of a neck-mounted AAM system to identify ovulation in lactating dairy cows between 7 to 30 DIM. This is the first study that has evaluated a neck-mounted sensor system to identify resumption of cyclicity in early lactation.

Overall, the sensor had 84.0% specificity and 34.1% sensitivity, indicating that the sensor is effective at detecting cows that are not cycling (i.e., anovular cows). Two previous studies assessed the performance of a neck-mounted (Valenza et al., 2012) or ear-attached (Schilkowsky et al., 2021) accelerometer system in lactating Holstein cows, past the VWP, that were treated with GnRH followed 7 d later by PGF to synchronize estrus. In the first study, 71% of cows were detected in estrus by the accelerometer system and 95% of cows showing estrus ovulated within 7 d after induction of luteolysis. Of the cows not detected in estrus by the accelerometer system, 35% ovulated within 7 d after induction of luteolysis. In the second study, 84% of cows were detected in estrus by the accelerometer system and 95% of cows showing estrus ovulated within 7 d after induction of luteolysis. Of the cows not detected in estrus by the accelerometer system, 62% ovulated within 7 d after induction of luteolysis. These results and our findings show that the sensitivity of an AAM system to detect ovulation in cows past the VWP is different compared with cows before the VWP.

Furthermore, cows with diseases postpartum tended to have greater proportion of anovular cows. The overall prevalence of anovulation in the present study was 35.9%. This agrees with a US survey including 8 herds (mean 23.3%; ranging from 7.3% to 41.7%; Bamber et al., 2009) and a Canadian survey including 17 herds (mean 19.5%; ranging from 5.0% to 45.0%; Walsh et al., 2007) where anovulatory status was determined using serial blood or milk P4 measurements. The overall prevalence of anestrus in the present study was 62.5%. Studies that also used an AAM system to evaluate the prevalence of anestrus observed a similar number of anestrous cows (Chebel and Veronese, 2020; Borchardt et al., 2021). Based on differences in the observation period and AAM system used, results are difficult to compare.

Inflammatory and metabolic diseases are major factors that underlie anovulatory conditions in dairy cows (Santos et al., 2016). Cows affected by any disease had a greater chance for anovulation compared with healthy cows. This agrees with a study by Santos et al. (2009) in which they compiled data from 8 experiments representing 5,719 cows from 7 farms. All cows were evaluated for cyclicity at 65 d postpartum by sequential P4 analysis in plasma 12 to 14 d apart. Calving-related disorders (e.g., dystocia, twins, stillbirth, and RFM) and diseases affecting the reproductive tract (i.e., metritis, clinical endometritis) were the major contributors for depressed cyclicity at d 65 postpartum.

As the reproductive efficiency of dairy cattle continues to improve in response to better management and the use of technology, novel reproductive management approaches will still be required to further optimize herd performance, profitability, and sustainability (Giordano et al., 2022). Targeted reproductive management is considered a novel approach to identifying subgroups of cows that might benefit from specific intervention to maximize their reproductive performance. Activity data within the VWP have been associated with reproductive performance (Borchardt et al., 2021; Bretzinger et al., 2023) and have already been used to enroll cows without estrus activity into timed AI protocols as part of a targeted reproductive management strategy (Rial et al., 2022; Gonzalez et al., 2023). Results from our study show that a neck-mounted AAM system can be better used to identify anovulatory cows than ovulatory cows. However, there was a high number of false-negative cows (i.e., cows that ovulated but had no estrus alert). A physiological explanation may be that the first ovulation postpartum is often accompanied without or weak estrus behavior and also followed by a short estrus interval (Crowe et al., 2014). It is assumed that high levels of estradiol during late gestation and parturition induce a refractory state to the estrogens present at the first postpartum ovulation. However, P4 from the corpus luteum secreted after the first ovulation seems to favor estrous expression during the next ovulatory cycle (Allrich, 1994). Also, priming of the hypothalamus with P4 by an increased number of estrus cycles before the first insemination may be associated with better responsiveness of estradiol receptors leading to improved estrus behavior (Thatcher and Wilcox, 1973).

Considering the low sensitivity of AAM systems, false-negative cows would be enrolled into complex protocols, although it might not be necessary. While it is unlikely that this has a detrimental effect on fertility, it might represent unnecessary costs for labor and hormones. Fertility of cycling cows subjected to an Ovsynch is about 35% when they start the protocol at a random stage of the cycle (Gümen and Seguin, 2003; Stevenson, 2008). One study enrolled cows to receive either their first AI using timed AI (Presynch-Ovsynch) or a strategic use of an AAM to inseminate cows via estrus while only using timed AI for cows not found in estrus (Burnett et al., 2017). The authors found that cows that were still anovular by 50 DIM had shorter days to pregnancy when inseminated using a timed AI protocol for the first AI postpartum, rather than by the AAM.

In conclusion, the results demonstrate that the sensor system had 84.0% specificity and 34.1% sensitivity within the VWP. The sensor's high specificity demonstrates its effectiveness in identifying cows that are not cycling (anovular cows). However, the relatively low sensitivity suggests that there were a significant number of false-negative alerts, meaning cows that ovulated but did not exhibit estrus behavior. This discrepancy might be due to the first ovulation often not being accompanied by estrus behavior and followed by a short estrus interval. Dairy cattle reproductive efficiency improves with better management and technology, and novel reproductive management approaches, such as targeted reproductive management, will be required to optimize herd performance, profitability, and sustainability. Overall, the study highlights the importance of accurately detecting ovulation and cyclicity in dairy cows to enhance reproductive management practices and maximize herd performance. Further research is needed to understand the physiological mechanisms behind false-negative alerts and improve the effectiveness of AAM systems in reproductive management strategies for dairy cattle.

## References

[bib1] Allrich R.D. (1994). Endocrine and neural control of estrus in dairy cows. J. Dairy Sci..

[bib2] Aungier S.P.M., Roche J.F., Duffy P., Scully S., Crowe M.A. (2015). The relationship between activity clusters detected by an automatic activity monitor and endocrine changes during the periestrous period in lactating dairy cows. J. Dairy Sci..

[bib3] Bach K.D., Heuwieser W., McArt J.A.A. (2016). Technical note: Comparison of 4 electronic handheld meters for diagnosing hyperketonemia in dairy cows. J. Dairy Sci..

[bib4] Bamber R.L., Shook G.E., Wiltbank M.C., Santos J.E.P., Fricke P.M. (2009). Genetic parameters for anovulation and pregnancy loss in dairy cattle. J. Dairy Sci..

[bib5] Borchardt S., Tippenhauer C.M., Plenio J.L., Bartel A., Madureira A.M.L., Cerri R.L.A., Heuwieser W. (2021). Association of estrous expression detected by an automated activity monitoring system within 40 days in milk and reproductive performance of lactating Holstein cows. J. Dairy Sci..

[bib6] Bretzinger L.F., Tippenhauer C.M., Plenio J.L., Heuwieser W., Borchardt S. (2023). Effect of transition cow health and estrous expression detected by an automated activity monitoring system within 60 days in milk on reproductive performance of lactating Holstein cows. J. Dairy Sci..

[bib7] Burnett T.A., Madureira A.M.L., Silper B.F., Fernandes A.C.C., Cerri R.L.A. (2017). Integrating an automated activity monitor into an artificial insemination program and the associated risk factors affecting reproductive performance of dairy cows. J. Dairy Sci..

[bib8] Chebel R.C., Veronese A. (2020). Associations between genomic merit for daughter pregnancy rate of Holstein cows and metabolites postpartum and estrus characteristics. J. Dairy Sci..

[bib9] Crowe M.A., Diskin M.G., Williams E.J. (2014). Parturition to resumption of ovarian cyclicity: Comparative aspects of beef and dairy cows. Animal.

[bib10] Dolecheck K.A., Silvia W.J., Heersche G., Chang Y.M., Ray D.L., Stone A.E., Wadsworth B.A., Bewley J.M. (2015). Behavioral and physiological changes around estrus events identified using multiple automated monitoring technologies. J. Dairy Sci..

[bib11] FASS (2020).

[bib12] Galvão K.N., Federico P., De Vries A., Schuenemann G.M. (2013). Economic comparison of reproductive programs for dairy herds using estrus detection, timed artificial insemination, or a combination. J. Dairy Sci..

[bib13] Giordano J.O., Sitko E.M., Rial C., Pérez M.M., Granados G.E. (2022). Symposium review: Use of multiple biological, management, and performance data for the design of targeted reproductive management strategies for dairy cows. J. Dairy Sci..

[bib14] Gonzalez T.D., Factor L., Mirzaei A., Montevecchio A.B., Casaro S., Merenda V.R., Prim J.G., Galvão K.N., Bisinotto R.S., Chebel R.C. (2023). Targeted reproductive management for lactating Holstein cows: Reducing the reliance on exogenous reproductive hormones. J. Dairy Sci..

[bib15] Gümen A., Seguin B. (2003). Ovulation rate after GnRH or PGF_2α_ administration in early postpartum dairy cows. Theriogenology.

[bib16] Hajian-Tilaki K. (2014). Sample size estimation in diagnostic test studies of biomedical informatics. J. Biomed. Inform..

[bib17] Lucy M.C. (2019). Symposium review: Selection for fertility in the modern dairy cow—Current status and future direction for genetic selection. J. Dairy Sci..

[bib18] McArt J.A.A., Nydam D.V., Oetzel G.R., Overton T.R., Ospina P.A. (2013). Elevated non-esterified fatty acids and β-hydroxybutyrate and their association with transition dairy cow performance. Vet. J..

[bib19] NRC (2001).

[bib20] Rial C., Laplacette A., Giordano J. (2022). Effect of a targeted reproductive management program designed to prioritize insemination at detected estrus and optimize time to insemination on the reproductive performance of lactating dairy cows. J. Dairy Sci..

[bib21] Roelofs J.B., Krijnen C., van Erp-van der Kooij E. (2017). The effect of housing condition on the performance of two types of activity meters to detect estrus in dairy cows. Theriogenology.

[bib22] Santos J.E.P., Bisinotto R.S., Ribeiro E.S. (2016). Mechanisms underlying reduced fertility in anovular dairy cows. Theriogenology.

[bib23] Santos J.E.P., Rutigliano H.M., Filho M.F.S. (2009). Risk factors for resumption of postpartum estrous cycles and embryonic survival in lactating dairy cows. Anim. Reprod. Sci..

[bib24] Schilkowsky E.M., Granados G.E., Sitko E.M., Masello M., Perez M.M., Giordano J.O. (2021). Evaluation and characterization of estrus alerts and behavioral parameters generated by an ear-attached accelerometer-based system for automated detection of estrus. J. Dairy Sci..

[bib25] Stevenson J.S. (2008). Progesterone, follicular, and estrual responses to progesterone-based estrus and ovulation synchronization protocols at five stages of the estrous cycle. J. Dairy Sci..

[bib26] Thatcher W.W., Wilcox C.J. (1973). Postpartum estrus as an indicator of reproductive status in the dairy cow. J. Dairy Sci..

[bib27] Valenza A., Giordano J.O., Lopes G., Vincenti L., Amundson M.C., Fricke P.M. (2012). Assessment of an accelerometer system for detection of estrus and treatment with gonadotropin-releasing hormone at the time of insemination in lactating dairy cows. J. Dairy Sci..

[bib28] Walsh R.B., Kelton D.F., Duffield T.F., Leslie K.E., Walton J.S., LeBlanc S.J. (2007). Prevalence and risk factors for postpartum anovulatory condition in dairy cows. J. Dairy Sci..

[bib29] Williams E.J., Fischer D.P., Pfeiffer D.U., England G.C.W., Noakes D.E., Dobson H., Sheldon I.M. (2005). Clinical evaluation of postpartum vaginal mucus reflects uterine bacterial infection and the immune response in cattle. Theriogenology.

